# Effect of Yoga Based Lifestyle Intervention on Patients With Knee Osteoarthritis: A Randomized Controlled Trial

**DOI:** 10.3389/fpsyt.2018.00180

**Published:** 2018-05-08

**Authors:** Singh Deepeshwar, Monika Tanwar, Vijaya Kavuri, Rana B. Budhi

**Affiliations:** Division of Yoga and Life Sciences, Swami Vivekananda Yoga Anusandhana Samsthana, Bengaluru, India

**Keywords:** knee osteoarthritis, integrative approach of yoga therapy (IAYT), handgrip strength (HGS), goniometer, falls efficacy scale (FES)

## Abstract

**Objective:** To investigate the effect of integrated approach of yoga therapy (IAYT) intervention in individual with knee Osteoarthritis.

**Design:** Randomized controlled clincial trail.

**Participants:** Sixty-six individual prediagnosed with knee osteoarthritis aged between 30 and 75 years were randomized into two groups, i.e., Yoga (*n* = 31) and Control (*n* = 35). Yoga group received IAYT intervention for 1 week at yoga center of S-VYASA whereas Control group maintained their normal lifestyle.

**Outcome measures:** The Falls Efficacy Scale (FES), Handgrip Strength test (left hand LHGS and right hand RHGS), Timed Up and Go Test (TUG), Sit-to-Stand (STS), and right & left extension and flexion were measured on day 1 and day 7.

**Results:** There were a significant reduction in TUG (*p* < 0.001), Right (*p* < 0.001), and Left Flexion (*p* < 0.001) whereas significant improvements in LHGS (*p* < 0.01), and right extension (*p* < 0.05) & left extension (*p* < 0.001) from baseline in Yoga group.

**Conclusion:** IAYT practice showed an improvement in TUG, STS, HGS, and Goniometer test, which suggest improved muscular strength, flexibility, and functional mobility.

CTRI Registration Number: http://ctri.nic.in/Clinicaltrials, identifier CTRI/2017/10/010141.

## Introduction

Osteoarthritis (OA) is the most common form of **arthritis** and leading cause of disability and loss of functions in the elderly population. It can affect any joints, but the knee is one of the most affected parts of the body in humans. There are several risk factors for OA such as obesity, smoking, intra-articular fractures, chondrocalcinosis, crystals in joint fluid/cartilage, female gender, prolonged immobilization, joint hypermobility, instability, peripheral neuropathy, prolonged occupational, or sports stress (1). The prevalence of knee osteoarthritis increases with age (2). Approximately 41.1% of males and 56.5% of females suffer from OA (3). Over 40% of adults between 50 and 75 years are affected with knee OA worldwide (4). The prevalence of knee OA in India is estimated to be 28.7% (5). A total of 11 COPCORD (Community Oriented Program for Control of Rheumatic Disorders) reports of knee OA data showed differences between rural (3.3%) and urban (5.5%) population of India (6, 7).

Symptoms of OA present as pain in and around the joints, morning stiffness, restricted joint movements associated with muscle weakness. Knee OA is associated with disrupted sleep, depression, increased sedentary behavior, less physical activity, obesity and decreased the quality of life (8). Bilateral knee osteoarthritis impaired the balance and increased the risk of fall, particularly in people with moderate knee osteoarthritis (9).

Non-pharmacological interventions such as exercise, Yoga, integrated approach of yoga therapy (IAYT), Tai-Chi, physiotherapy, acupressure, naturopathy, and massage therapy showed improvements in quality of life along with a reduction in pain, improved physical functions, psychological balance in patients with knee OA (10–14). These non-pharmacological rehabilitation interventions have focused mainly on practices for Knee OA that produces only small to moderate benefits with the limited durability of effects on the symptoms of knee OA (15). These interventions provided substantial benefits, but are underutilized, and the efficacy and safety remain poorly defined. In few earlier studies, yoga showed promising changes in reducing pain, morning stiffness, and increased flexibility, muscular strength and overall quality of life in knee OA patients (16–19).

Yoga, a mind-body intervention, originated in India. Different schools of yoga (such as Iyengar yoga, IAYT, hatha yoga, etc.) developed a therapeutical intervention for knee OA. A pilot study was conducted on nine participants, using modified Iyengar yoga postures (90-min classes once in a week for 8 weeks) as a treatment modality and showed potential reductions in pain and disability caused by knee OA (20). Few other studies compared yoga therapy with different interventions such as traditional stretching and strengthening exercises or no structured group exercise for 6 weeks and showed functional changes and improvement in the quality of life in traditional practice and yoga-based approach (21). Ebnezar et al. investigated transcutaneous electrical stimulation and ultrasound treatment followed by IAYT intervention (40 min) and reported that IAYT is better than physiotherapy exercises for reducing pain, morning stiffness, state and trait anxiety, blood pressure and pulse rate in OA patients (18). This study was limited to numerical pain scale and state and trait anxiety (STAI 1&2). Meta-analyses of yoga for musculoskeletal problems suggest that yoga is helpful for chronic pain and low back problems in older women population (16).

Studies on aerobic exercises including physical activities, yoga, and Tai Chi, have long been a rehabilitation intervention for treating patients with OA in decreasing pain, joint tenderness and improving functional status (22). Most of these studies indicate the long-term effect of yoga on symptoms of OA. There are very few studies reporting the immediate impact of yoga on patients with knee OA and self-efficacy. Also, limited data are available to support the efficacy of IAYT intervention for OA management. Few studies have methodological issues related to their design such as small sample size, wide-ranging age group due to unavailability of similar age group participants, whereas other studies have a long-term intervention, high attrition rate, etc.

Hence, the present study intended to investigate the immediate effect of 1-week integrated approach of yoga therapy (IAYT) intervention in older adults with knee OA. We hypothesized that a brief intervention of yoga practice may have a positive effect on, (i) Falls Efficacy Scale (FES), (ii) handgrip strength (HGS), (iii) Timed up and Test (TUG), (iv) Sit to Stand (STS), and (v) knee extension and flexion.

## Materials and methods

### Participants

**Figure 1:**
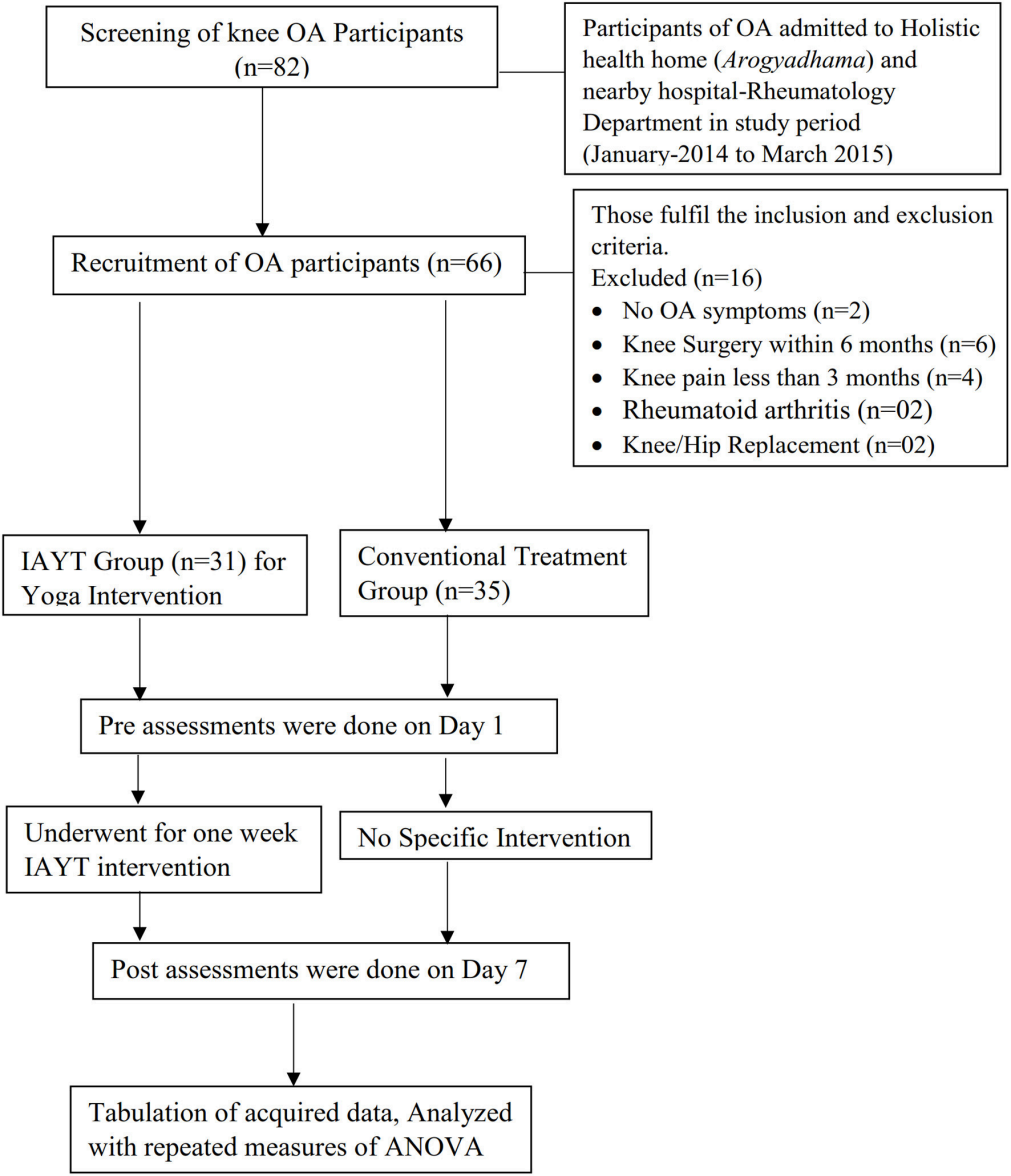
CONSORT Flow diagram of study participants.

A total of 66 participants (50 female and 16 male) aged 30–75 years (60.2 ± 8.2 years), were recruited from *Arogyadhama*, a home-based health center, S-VYASA, Bangalore and the nearby area between April 2015–July 2015. The sample size was calculated with G-Power software by fixing the alpha at 0.05 powered at 0.8 and an effect size of 0.71 based on the mean and *SD* of an earlier study (18). The inclusion criteria were, patients suffering from knee OA for more than 3 months (diagnosed by a physician), fully ambulant, literate, and willing to participate in the study. Patients with rheumatoid arthritis, autoimmune diseases, malignancies, knee surgery or knee-arthroscopy, and knee pain caused by congenital dysplasia were excluded. The flow chart of study participants from enrollment to completion is given in Figure 1.

Prospective participants were informed about the trial through media reports and advertisements in local newspapers and University magazine. The CONSORT Flow diagram of the trial is given in Figure 1 and the demographic details collected for all participants (age, gender, duration of OA complaints, BMI, education, and Occupation) are given in Table 1.

**Table 1:** Characteristics of the study participants (*n* = 66).

**Participants**	**Yoga (*n* = 31)**	**Control (*n* = 35)**
Gender—50 Females (75%)		
Males	6 (61.83 ± 9.1 years)	10 (60.13 ± 8.6 years)
Females	25 (59.8 ± 8.2 years)	25 (59.4 ± 9.4 years)
Duration of pain, in years; mean (SD)	2–16 years; 11.52 (4.01)	2–14 years; 12.31 (5.36)
BMI (mean; *SD*)	28.15 (5.80)	30.02 (4.15)
Education: *n* (%)		
(<6 years)	22 (71%)	19 (54%)
(>6 and 12 years)	7 (22%)	12 (34%)
(>12 years)	2 (7%)	4 (11%)
Occupation: n (%)		
Housewife	18 (58%)	24 (69%)
Govt. Employee	2 (5%)	1 (3%)
Private Employee	7 (20%)	8 (23%)
Retired	5 (17)	2 (5%)

#### Ethical consideration

The research study was approved by the Institutional Review Board (IRB) of the S-VYASA University (No. SVYASA/MSc/IRB/10/21) and conducted under the guidance of senior doctors and therapists of the University. All subjects were informed about the trial, and written informed consent was obtained from the participants of this study.

#### Design

Recruited participants were randomly divided into two groups, i.e., Yoga group with the intervention of integrative approach for yoga therapy (IAYT) and Control group without any form of yoga intervention. The study set-up was completely a yoga-based lifestyle where all participants in yoga group followed intervention of IAYT treatment for 6 days. All assessments and treatment plans for participants were discussed with senior doctor and therapist. All participants in both groups continued their medication as per the requirements. It was not possible to mask the yoga intervention from the subjects. However, the investigators who collected primary and secondary outcomes were blind to the intervention (23).

### Intervention

#### Integrated approach of yoga therapy (IAYT)

IAYT module for arthritis was developed using a holistic approach to health management at physical, mental, emotional, and intellectual levels (24). The practices were yoga postures (*asana*), yoga breathing (*pranayama*), relaxation techniques, meditation and lectures on yogic lifestyle, devotional sessions, and stress management through yogic counseling. The yogic practices for knee OA included simple yogic movements and postures that provided stretching, flexibility, strengthening weak muscles and relaxation of body and mind (24). Yogic breathing helps participants to achieve a slow rhythmic pattern of breathing with slowing down the breathing pattern, deep inhalation, and longer exhalation as the foundation (25). Cyclic meditation, Om meditation and devotional sessions (*prayers)* are part of meditation to control the surge of negative emotions. Lectures and individual yogic counseling for stress management were effectively focused on knee pain (26).

Cleansing techniques (*Kriyäs*) were used to help clean and refresh the optical path, respiratory tract, and gastrointestinal tract. All participants practiced intense candlelight gazing (*Trāṭaka*), nasal cleansing with water and catheter (*Jala and sutra neti*), frontal brain cleansing breath (*Kapālabhāti*), vomiting with lukewarm saline water (*Vamana dhauti*), partial colon cleansing (*Laghuśankha Prakshālana*) (27). These techniques were done everyday twice (morning and evening) during the intervention period of 7 days. The summarized yoga practices for knee OA are given in Table 2.

**Table 2:** Yoga practices module for knee OA.

**S. No**.	**Practices**	**Practice name (Sanskrit and English)**	**Duration of practice**
1.	Breathing practices	Hands in and out Breathing	5 rounds (2 min)
		Hands Stretch Breathing	5 rounds (2 min)
		Ankle Stretch Breathing	5 rounds (2 min)
2.	Loosening practices in standing	Twisting	5 rounds (2 min)
		Side bending	5 rounds (2 min) on each side
3.	Loosening practices in sitting	Knee Cap Tightening	5 rounds (2 min) each, both legs
		Passive Patella Movement (Up and Down, In and Out, Rotation)	10 rounds (4 min) both legs
		Knee bending	5 rounds (2 min) each, both legs
4.	Loosening practices in Supine	Folded Leg Lumber Stretch (Left, Right, Both)	5 rounds (2 min)
		Cycling	5 rounds (2 min) both legs
		Straight Leg Raising (Left, Right and Both)	5 rounds (2 min)
5.	Yoga Posture- Sitting	*Paschimottasana* (Seated forward bend Pose)	3 rounds (3 min)
		*Bhūṇamanāsana* (Earth Salutation Pose)	3 rounds (3 min)
6.	Yoga Posture-Prone	*Bhujaṅgāsana* (Cobra Pose)	3 rounds (3 min)
		*Salabhāsana* (Locust Pose)	3 rounds (1 min)
		*Vipareetkarani* (Inverted Pose)	2 min
7.	Yoga posture- Supine	*Setubandhāsana* (Bridge Pose)	2 rounds (1 min)
		*Markaṭāsana* (Lumbar Stretch Pose)	2 rounds (1 min)
		*Savāsana* (Corpse Pose)	5 min
8.	Relaxation Techniques	Instant Relaxation Technique	2 min
		Quick Relaxation Technique	5 min
		Deep Relaxation Technique	10 min
9.	Kriyas (Cleansing techniques)	*Jalaneti* (Nasal Cleansing with Water)	30 min
		*Vamanadhouti* (Internal Cleansing by Water)	15 min
		*Trāṭaka* (Candle Light Gazing)	10 min
		*Kapālabhāti* (Frontal Brain Cleansing)	5 min
10.	*Prāṇayama* (Yoga Breathing)	*VibhāgīyaPrāṇayama* (Sectional Breathing)	3 rounds (3 min)
		*Nādīśuddhī* (Alternate Breathing)	9 rounds (3 min)
		*Brahāmarī* (Humming Bee Breathing)	9 rounds (3 min)
		*Bhastrikā* (Bellows Breathing)	9 rounds (3 min)
11.	Cooling *Prāṇayama*	*Śītali* (Rolling Tongue Breathing)	9 rounds (3 min)
		*Śitkārī* (Folded Tongue Breathing)	9 rounds (3 min)
		*Sadantā* (Clenched Teeth Breathing)	9 rounds (3 min)
12.	Meditation	*Nādānusandhāna* (A,U,M and A-U-M Kara chanting)	10 min
		Om Meditation	10 min
		Cyclic Meditation	30 min
		Mind Sound Resonance Technique	10 min

### Outcome measures

All participants were assessed for primary and secondary outcomes twice, at baseline (day 1) and end of study period, day 7.

#### Primary outcomes

**(i) Timed up and go Test (TUG)**—TUG is an easy and low-cost test developed to assess the functional mobility of patients during everyday activities. The test comprises the following sequence of movements: to stand up from a standard chair, walk 3 m, turn, walk back to the chair and sit down again. The time taken by patients to complete the sequence of this movement is recorded and compared before and after treatment (28). The internal consistency (Cronbach's alpha) was 0.74.

**(ii) Sit-to-Stand (STS)**—Participants were instructed to stand up five times from a chair without using the support of their arms, as fast as possible. The test was repeated twice as this improved the reliability of the test, and the average time will be calculated in seconds (29). The correlation coefficients of intra-session reliability and test-retest reliability were 0.95 and 0.99, respectively. The convergent validity of the five-repetition sit-to-stand test was supported by significant correlation with a one-repetition maximum of the loaded sit-to-stand test, isometric muscle strength, scores of Gross Motor Function Measure, and gait function (r or rho = 0.40–0.78) (30).

**(iii) Goniometer test** for flexibility and range of motion—Participants were seated on a chair with legs stretched in front called Right and Left Extension. The goniometer was placed on the knee and was asked to bend the leg at the knee as far as they could, Right and Left Flexion, and the degree of the bend was measured with the goniometer. The average range of motion (ROM) of the knee is 120–150° (31). The data analysis revealed that the inter-tester reliability (*r* = 0.98; ICC = 0.99) and validity (*r* = 0.97–0.98; ICC = 0.98–0.99) were high (32). An overall mean score was calculated for each participant.

#### Secondary outcomes

**(i) Handgrip Strength Test (HGS)**—Handgrip strength of both hands right handgrip strength (RHGS) and left handgrip strength (LHGS) were assessed using a handgrip dynamometer. Subjects were tested in 6 trials, 3 for each hand alternately, with a gap of 10 s between trials (33).

**(ii) Falls Efficacy Scale (FES)**—FES is an instrument to measure fear of falling, based on the operational definition of this fear as “low perceived self-efficacy at avoiding falls during essential, non-hazardous activities of daily living.” FES is a 10-item rating scale with test–retest reliability (*r* = 0.71), used to assess confidence in performing daily activities without falling (34). Each item is rated from 1 = extreme confidence, to 10 = no confidence at all. Participants who reported avoiding activities because of fear of falling had higher FES scores, representing lower self-efficacy or confidence than those not reporting fear of falling.

### Procedure

All recruited participants were randomized in two groups, i.e., Yoga group (*n* = 31; 59.8 ± 10.21 years) and Control group (*n* = 35; 61.07 ± 9.17 years), using systematic sampling method. The data collected of 66 participants on Day 1 and Day 7 were extracted with the help of the therapist from rheumatology department. There were no dropouts in this study, and all collected data were observed carefully. The data were tabulated and no missing values were found.

Data were obtained from participants as per the stipulated instructions in the manuals of questionnaires and tests.

## Data analysis

Statistical analysis was carried out using the Statistical Package for the Social Science (SPSS version 20.00, IBM Corp., USA). The scores were assessed for between-group differences in the change of outcome measures, i.e., Timed up and test (TUG), Sit to stand test (STS), Handgrip strength test (HGS), Extension and Flexion, and Falls Efficacy Scale (FES) after the 1-week intervention of IAYT.

Within Group and Between-Group comparisons were performed for exploratory reasons and are given in Table 3. Test of normality showed no significant difference in age, duration of osteoarthritis and socio-economic status between the groups. Repeated measures of Analysis of Variance (ANOVA) were performed for each outcome measures with two factors: (1) Groups: Yoga and Control; and (2) number of assessments: Pre and Post. Repeated measures of ANOVA were carried out separately followed by *post-hoc* analysis with Bonferroni correction, for two-time points of all the outcome measures. All comparisons were made between pre and post mean values of each outcome measure. If the *p*-value was *p* ≤ 0.05, the results were considered statistically significant.

**Table 3:** Comparison of change in primary and secondary outcomes in IAYT and control groups.

**Variables**	**Within group**				**Between-Group (*p*-value)**	**2-Way repeated measures of ANOVA**		
	**Yoga group**		**Control group**			**F group (*p*-value)**	**F time (*p*-value)**	**F interaction (*p*-value)**
	**Before**	**After**	**Before**	**After**				
**PRIMARY OUTCOMES**								
Timed Up and Go Test (TUG)	19.16 ± 5.99	15.57 ± 5.23^***^	18.56 ± 6.41	19.02 ± 5.19^*^	0.014	2.500	35.413^$$$^	8.842^$$^
Sit-to-Stand (STS)	18.35 ± 6.25	14.22 ± 4.65^***^	19.28 ± 5.69	18.06 ± 5.71	0.004	4.72^$^	30.973^$$$^	9.092^$$^
Goniometer -	177.58 ± 3.63	179.36 ± 2.14^*^	178.17 ± 3.25	176.21 ± 3.05	NS	0.912	6.298^$^	1.142
*(i) Right Extension*								
*(ii) Right Flexion*	45.52 ± 13.03	37.23 ± 11.28^***^	46.22 ± 13.40	44.54 ± 14.32	0.026	2.38^$^	28.910^$$$^	12.671^$$$^
*(iii) Left Extension*	176.52 ± 4.49	179.32 ± 1.90^***^	175.84 ± 4.22	176.20 ± 5.19	0.035	0.887	12.429^$$^	7.927^$$^
*(iv) Left Flexion*	46.39 ± 15.47	39.84 ± 12.55^***^	47.89 ± 16.24	45.43 ± 15.12^*^	NS	1.96	31.435^$$$^	6.488^$^
**SECONDARY OUTCOMES**								
Falls Efficacy Scale (FES)	39.13 ± 10.36	41.58 ± 11.18	37.53 ± 11.05	36.21 ± 13.14	NS	0.258	2.180	2.081
Hand Grip Strength (HGS)- *(i) Right Hand Grip Strength (RHGS)*	22.42 ± 6.28	23.67 ± 6.05	24.51 ± 7.35	24.34 ± 5.75	NS	0.886	0.937	1.779
*(ii) Left Hand Grip Strength (LHGS)*	21.11 ± 6.32	22.55 ± 6.88^**^	22.11 ± 6.01	20.2 ± 6.32	NS	0.821	7.625^$$^	1.082

* p < 0.05;

** p < 0.01;

*** *p < 0.001 significant difference before and after yoga intervention*.

$ < 0.05;

$$ < 0.01;

$$$ *< 0.001 showed main effect or interaction effect in 2-way repeated measures ANOVA*.

## Results

The demographic data of recruited participants are given in Table 1. The repeated measures of ANOVA were performed for each outcome measure with two factors, i.e., groups (Yoga and Control) and times of assessment (Pre and Post). The primary and secondary outcome scores of within-group analysis are shown in Table 3. The 2-way ANOVA results showed that there was a significant interaction between “group” and “time” of (i) TUG (*F* = 8.84; *p* < 0.01), (ii) STS (*F* = 9.09; *p* < 0.01), and (iii) Goniometer (a) Right Flexion (*F* = 12.67; *p* < 0.001), (b) Left Extension (*F* = 7.93; *p* < 0.01), and (c) Left Flexion (*F* = 6.49; *p* < 0.05). *Post-hoc* analysis with Bonferroni correction showed significant decrease in TUG (*p* < 0.001), STS (*p* < 0.001), increased Right & Left Flexion (*p* < 0.001) and Right (*p* < 0.05) & Left (*p* < 0.001) extension in *primary outcomes*.

**Figure 2:**
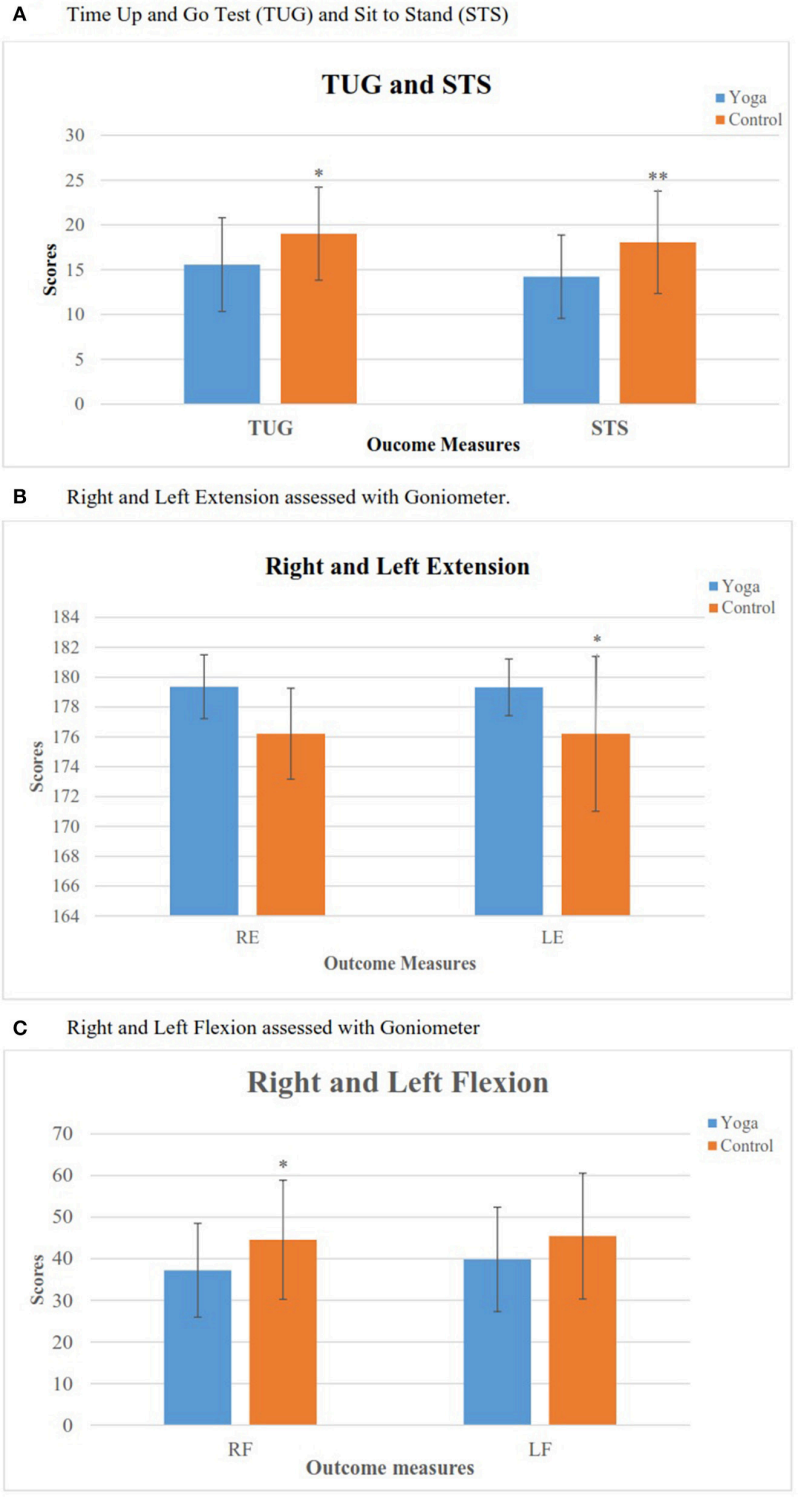
Comparison between yoga and control groups after intervention. **(A)** Time Up and Go Test(TUG) and Sit To Stand (STS). **(B)** Right and left extension assessed with Goniometer. **(C)** Right and left flexion assessed with Goniometer. ^*^p < 0.05, ^**^p < 0.01.

Whereas in the *secondary outcomes*, handgrip strength showed significant increase in LHGS (*p* < 0.01) in yoga group after 1 week IAYT intervention and no changes falls efficacy score. In Control group, we observed there was no relief of symptoms. After 1 week, we observed that the Control group had a significant increase in TUG (*p* < 0.05) and a decrease in Left Flexion (*p* < 0.05) suggesting worsened symptoms after 1 week with conventional treatment alone. Between group analysis showed there was significant difference in post assessments of TUG (*p* < 0. 05), STS (*p* < 0.01), Right Flexion (*p* < 0.05), Left Extension (*p* < 0.05) of Yoga and Control group as shown in Figures 2A–C.

## Discussion

The results of the present study of the 1-week integrated approach of yoga therapy (IAYT) demonstrated significant improvements in TUG and STS tests in the Yoga group and no changes were observed in Control group. Yoga group participants reported significantly shorter time taken to perform different physical tests after 1-week yoga intervention which suggest better functional performance. The TUG test assesses multiple components of balance and mobility (35). The STS movement is one function people frequently use as they change from a sitting position to a standing position. STS requires forward movement of the center of mass while still seated (in preparation to stand), acceleration of the center-of-mass both in the anterior, posterior, and vertical plane, push off and stabilization once standing is achieved (36, 37). This movement is defined as a transitional movement to the upright posture requiring movement of the center of mass from a stable position to a less stable position over extended lower extremities (38). The HGS is a reliable measurement when standardized methods and calibrated equipment are used, even when there are different assessors or different brands of dynamometers (39, 40). Grip strength is related to the predictive of other health conditions. In the present study, right and left handgrip strength showed improvement after 1 week IAYT intervention. Previous studies reported that handgrip strength is positively related to normal bone mineral density in postmenopausal women (41), and can be used as a screening tool for women at risk of osteoporosis (42).

Additionally, in the present study, Yoga group patients showed that there was a significant decrease in knee pain and stiffness, and significant improvement in mobility, measured through right and left leg extension and flexion test. These results are consistent with previous findings of Schilke et al. were 10 patients with knee pain, have undergone 8 weeks of the isokinetic muscle-strength-training program and showed a significant decrease in pain and stiffness. There was also a significant decline in arthritis activity after intervention and an increase in all strength measures of right-left flexion and left-leg extension across the training period (14).

One study on rheumatoid arthritis (RA) patients aged 18 years and older, underwent 8 weeks of yoga (two 60-min classes and one home practice/wk) reported higher physical component summary (PCS), walking capacity, positive affect and lower center for epidemiologic studies depression scale. Improvements were also shown in SF-36 health-related quality of life, role physical (work and daily activity impairment due to physical health), pain, general health, vitality, and mental health scale (43). Yoga showed a reduction in pain, depression and more significant improvement in life satisfaction after intervention (44). Yoga is mind-body interventions, that impart stress management with physical activity may be well suited for osteoarthritis and rheumatoid arthritis. Another therapeutic intervention of Iyengar yoga in patients with knee OA, EMG biofeedback showed a significant reduction in pain and improvement in functional ability (19). This suggests that yoga along with conventional therapy provides better results in chronic knee osteoarthritis regarding pain and functional disability. In a comparison of conventional therapy and add-on yoga for 56 patients of knee rehabilitation after total knee arthroplasty showed that there was a significant change for pain, stiffness and functional subscales of Western Ontario and McMaster Universities OA Index (WOMAC) Scale in both the groups (45). This indicates that yoga *asana* protocol works better than physiotherapy alone. The practice of yoga is doable, easy to follow, safe and most important is useful for patients with knee OA. There is evidence that Tai-Chi and yoga are safe and showed significant reduction of pain and improvement of physical function and quality of life in patients (46). The physical posture (*hatha* yoga) practice also helps to reduce pain and symptoms of OA and increase scores of daily activities, sports, spare-time activities, and quality of life (17). The practice of yoga effects on knee OA reported positive outcomes on symptoms including pain, flexibility, functional disability, anxiety, and quality of life (20). Earlier studies indicated subsided pain intensity in walking scale and improvement in WOMAC and quality of life after yoga practice. The resting pain and morning stiffness were studied in a former study, and the current study is a continuation of the same intervention (i.e., IAYT). The present study reported reduction in Time Up and Go test, Sit To Stand test. These results show that yoga practice improves muscular strength, better movement, and flexibility.

The possible mechanism of yoga therapy-related changes in symptoms of OA is not known. The multifactorial approach of yoga therapy includes physical postures (*asanas*), breathing practices (*pranayama*), meditation (*dhyana*), spiritual and emotional cultures discourses may help to the amelioration of OA symptoms. Yoga therapy intervention may increase cartilage proteoglycan content and prevent cartilage degeneration (47). This is helpful for the strengthening of periarticular muscles (i.e., quads and hamstrings) that normally contract to stabilize the knee joint pain. Also, yoga practice may prevent synovial fluid volume deterioration by stretching and strengthen different parts of the body, massaging and bringing fresh blood to the internal organs while rejuvenating the nervous system and lubricating the joints, muscles, and ligaments. It is purported to have different effects on the nervous and circulatory systems, coordination and concentration and calming effect on the body. This also suggests that yoga practice helps in reducing several psychological factors such as stress, anxiety, depression, mood disturbances, and enhance self-esteem and quality of life (43) in individuals with chronic pain and arthritic conditions (48). It can be concluded that yoga can be used as a complementary treatment along with conventional treatment to improve the situation of people with knee osteoarthritis.

There are several limitations to this study such as very short duration of yogic intervention, confounding variables such as diet, controlled environment, small sample size, etc. Another limitation was no standard self-reported measure of osteoarthritis symptoms was used. Further, sample was not balanced by gender, so the outcome of the study cannot be generalized and future study can be planned with equal number of both the gender. Additionally, placebo is reported to be effective for OA, especially for subjective outcomes such as pain, stiffness, self-reported function, and physician global assessment and in the current study no placebo group was taken. Hence, the placebo effect on the outcome cannot be ruled out in this study and further study can be planned with placebo group. The strength of the present study is its cost-effectiveness and use of non-invasive intervention and assessments. This study comprises intensive lifestyle modification program with self-corrective practice. The IAYT intervention could be used as an add-on treatment of alternative and complementary therapy for osteoarthritis.

## Conclusion

In summary, the previous evidence and present study suggest that yoga is an acceptable and safe intervention, which may result in clinically relevant improvements in pain and functional outcome associated with a range of musculoskeletal conditions such as muscular dystrophy, osteoarthritis, rheumatoid arthritis, etc. The present study would suggest that 1 week of IAYT may be useful in decreasing pain and increasing functional mobility in these patients over time.

## Author contributions

SD helped in trial design, allocated participants, collected the data, analysed, interpreted data, and wrote the manuscript; MT performed the literature search and evaluated the outcomes (blind assessor); VK data analysis; assisted in manuscript compilation and RB editing; assisted in manuscript compilation and correspondence.

### Conflict of interest statement

The authors declare that the research was conducted in the absence of any commercial or financial relationships that could be construed as a potential conflict of interest.
